# Higher amoebic and metronidazole resistant forms of *Blastocystis* sp. seen in schizophrenic patients

**DOI:** 10.1186/s13071-022-05418-0

**Published:** 2022-09-05

**Authors:** Freddy Franklin, Arutchelvan Rajamanikam, Chandramathi Samudi Raju, Jesjeet Singh Gill, Benedict Francis, Luke Woon Sy-Cherng, Suresh Kumar

**Affiliations:** 1 Department of Parasitology, Universiti Malaya (UM), Kuala Lumpur, 50603 Malaysia; 2 Department of Medical Microbiology, Universiti Malaya (UM), Kuala Lumpur, 50603 Malaysia; 3 Department of Phycological Medicine, Pusat Perubatan Universiti Malaya (PPUM), Kuala Lumpur, 50603 Malaysia; 4 Department of Psychiatry, Pusat Perubatan Universiti Kebangsaan Malaysia (PPUKM), Kuala Lumpur, 50603 Malaysia

**Keywords:** *Blastocystis*, Schizophrenia, Amoebic, Metronidazole resistant

## Abstract

**Background:**

*Blastocystis* sp. is one of the most common colonisers of the intestinal tract that demonstrate strong interaction with accompanying gut bacteria. Previously, the protozoan isolated from individuals with irritable bowel syndrome (IBS) showed altered phenotypic features suggesting that it can be triggered to become pathogenic. Previous studies reported altered gut microbiota and high prevalence of *Blastocystis* sp. in schizophrenia patients. However, the phenotypic characteristics of *Blastocystis* sp. isolated from individuals with SZ have yet to be described.

**Methods:**

In this study, faecal samples from 50 patients with severe schizophrenia (SZ) and 100 non-schizophrenic (NS) individuals were screened for *Blastocystis* sp. infection. Positive isolates were subjected to genotypic and phenotypic characterization.

**Results:**

We found that 12 out of 50 (24%) SZ and 5 out of 100 (5%) NS individuals were detected *Blastocystis* sp. positive using both in vitro culture and PCR method with no significant association to age and gender. Out of the 15 sequenced isolates, ST3 was the most prevalent subtype (66.7%) followed by ST1 (20%) and ST6 (13.3%). The isolates from SZ individuals demonstrated significant slower growth rate (34.9 ± 15.6 h) and larger range of cell diameter (3.3–140 µm). We detected higher amoebic forms and metronidazole resistance among SZ isolates with variation in cell surface glycoprotein where 98% of cells from SZ showed consistent medium to high binding affinity (+ 2 to + 3) to Concavalin A staining compared to NS isolates that demonstrated only 76% high lectin (+ 3) binding affinity. Cysteine and serine protease levels were predominantly found among SZ isolates. We also demonstrate the presence of metalloprotease in *Blastocystis* sp. especially among NS isolates. Introduction of solubilised antigens from SZ isolates increased the cell proliferation of HCT116 cells by two fold when compared to NS isolates.

**Conclusion:**

Our findings demonstrated *Blastocystis* sp. isolated from SZ individuals showed variation in phenotype specifically in morphology and drug resistance. The findings indicate that the gut environment (SZ and NS) and treatment of SZ could have influenced the phenotype of *Blastocystis* sp.

**Graphical Abstract:**

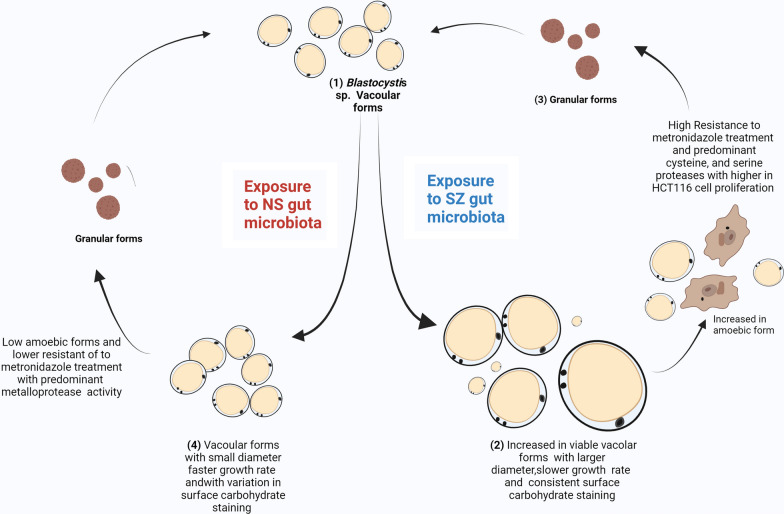

**Supplementary Information:**

The online version contains supplementary material available at 10.1186/s13071-022-05418-0.

## Background

*Blastocystis* sp. is the most prevalent gut protozoan found in stools. The prevalence of this microorganism is reported to be around 0.5–23% [1, 2] in developed nations whereas in third world countries the prevalence is around 22.1–100% [3, 4]. *Blastocystis* sp. is a polymorphic organism and predominantly exists as vacuolar, granular, amoebic, and cyst forms in the intestinal tract [5, 6]. It is commonly associated to non-specific gastrointestinal symptoms such as mild diarrhoea, flatulence, weight loss, and abdominal cramps. Infected individuals who do not manifest such symptoms are commonly known as asymptomatic carriers [7].

The controversy about whether *Blastocystis* sp. is a pathogen remains a debate. In the past decade, several studies have reported potential pathogenicity and virulence among certain subtypes of *Blastocystis* sp., specifically ST1 [8, 9], ST3 [10, 11],ST4 [12] and ST7 [13, 14]. The characteristics attributing to a microorganism being a pathogen include the type of surface protein [15], ultrastructural forms [16, 17], amoebic formation [18, 19], and protease activity [20, 21]. Although *Blastocystis* sp. from different microbial environment have shown morphological differences [22], this association remains inconclusive. There is a need to collect more data to establish a conclusive association.

Schizophrenia(SZ) is a neuropsychiatric disorder that affects 0.5–1% of the human population worldwide [23]. The incidence rate of SZ in Malaysia has been reported to be around 0.4% [24]. The aetiology of SZ is not well understood and is often attributed to multiple factors such as genetics and environment, leading to brain abnormality [25, 26]. Previously, parasitic infections such as *Toxoplasma gondii* have been established as a risk factor of SZ [27]. Lately, the focus has shifted towards the gut microbiome and its ability to affect the brain chemistry through the gut brain axis. The gut microbiome of SZ individuals has been reported to be different from that of healthy individuals with many studies reporting intestinal dysbiosis among SZ individuals [28]. While growing literature of NGS studies reported no differences of α-diversity among SZ individuals when compared to controls, there were significant differences in display in terms of β-diversity among SZ with different abundances of specific bacterial genera such as *Prevotella* and *Bacteroides* to name a few [29, 30]. Most recently, a study reported 55% prevalence of *Blastocystis* sp. infection among hospitalised male schizophrenic patients in Iran [31] although the prevalence in the general population is only 3.33–23.6% [32]. The prevalence data of *Blastocystis* sp. in SZ patients in Malaysia are scarce. Moreover, the characteristics of *Blastocystis* sp. isolated from these patients with disparate gut microbiota profiles are yet to be characterised.

In this study, we investigated the prevalence of *Blastocystis* sp. in chronic SZ patients in caring homes in urban Selangor, Malaysia, and evaluated the phenotypic and genotypic differences of *Blastocystis* sp. compared with NS individuals.

## Methods

### Cohort for sample collection

Participants were evaluated and diagnosed based on the Structured Clinical Interview (SCID) and the Diagnostic and Statistical Manual of Mental Disorders, fourth edition, text revision DSMIV-TR (by a certified psychiatrist), and grouped based on the Clinical Global Impression Scale (CGI). Patients that have taken any anti-protozoal and antibiotic medications 2 to 3 weeks prior to recruitment are excluded from the study. Ethical approval for collection of stool samples was obtained from the Medical Ethics Committee of University Malaya Medical Centre (UMMC), Kuala Lumpur, Malaysia (20191226-8107), and Research Ethics Committee of the National University of Malaysia (UKM PPI/111/8/JEP-2020-725) according to the Declaration of Helsinki. A written consent was obtained from all the participants prior to sample collection. A series of questionnaires comprising questions of standard demographic questions as well as questions regarding presence of gastrointestinal symptoms was administered before the sample collection. A total of 50 stool samples were collected randomly from patients with chronic SZ (CGI 7) from four different caring homes located in Petaling Jaya, Selangor. All patients were out-patients of PPUM and PPUKM and were on antipsychotic drug treatment during the stool sample collection. In addition, a total of 100 stool samples were collected from volunteers with similar mean age group attending PPUM for a range of other illnesses and was used as an internal control, non-schizophrenic (NS). These volunteers were verified as having no history of schizophrenia illness/symptoms (CGI 0) or any drug usage by a series of questionnaires and patient medical files by their respective doctors.

### Screening, isolation, and cultivation of *Blastocystis* sp.

Stool samples were screened for *Blastocystis* sp. infection using xenic culture method (XCD) involving in vitro culture in Jones medium [33] and conventional polymerase chain reaction (PCR) technique. A pea-sized (≈250 mg) stool sample was cultured in Jones medium (JM) supplemented with 10% horse serum (Gibco Laboratories, Life Technologies, New York, USA) and incubated at 37 °C. The cultures that were positive for *Blastocystis* sp. were sub-cultured and maintained every 3 days for at least up to 1 month before analysing its phenotypic characteristics.

### Faecal genomic DNA extraction and PCR screening

A pea-sized (≈250 mg) stool sample was subjected to DNA extraction using QIAamp Power Fecal Pro DNA kit (QIAGEN, Hilden, Germany) according to the manufacturer’s instruction. DNA extracted was quantified and analysed for purity using Nanodrop and agarose gel electrophoresis. A 20-μl reaction volume containing 18 μl of master mix from KAPA (ROCHE, Mannheim, Germany), 1 µl of template DNA, and 0.5 µl (10 µM) of forward and reverse primers, BL18SPPF1 5′–AGTAGTCATACGCTCGTCTCAAA–3’ and BL18SR2PP 5’–TCTTCGTTACCCGTTACTGC–3’, respectively, were used for the screening of *Blastocystis* sp., adapting the protocol from a previously established study [34]. The amplicons, ranging from 320–342 bp depending on the subtype, were sent for sequencing for further species confirmation.

### DNA extraction for genetic analysis of *Blastocystis* sp. isolates

Day 3 cell culture sediment was washed thrice with phosphate buffer saline (PBS) (Sigma-Aldrich, St Louis, MO, USA) and reconstituted to a final volume of 250 µl. Total DNA was extracted and purified using InstaGene Matrix (Bio-Rad Laboratories, CA, USA) according to manufacturer’s protocol. The extracted DNA template was used for subtyping using conventional PCR with primers BhRDr 5’–GAGCTTTTTAACTGCAACAACG–3’ and RD5 5’–ATCTGGTTGATCCTGCCAGT–3’ to generate amplicons of the 600-bp region of the *SSU*-rRNA gene of *Blastocystis* sp. [35, 36]. Online tools (https://pubmlst.org/bigsdb?db=pubmlst_blastocystis_seqdef&page=sequenceQuery) were used to further assign the alleles. All the sequences generated from primer pair BhRDr and RD5 were deposited in GenBank with accession numbers ON564757-ON564771.

### Growth profiling of *Blastocystis* sp.

Day 3 cultures of each *Blastocystis* sp. isolate were pooled and washed with 1× PBS. A total of 1 × 10^5^ of *Blastocystis* sp. cells was inoculated in microcentrifuge tubes containing JM supplemented with 10% horse serum bringing the final volume to 1 ml and incubated at 37 °C up to 10 days. All experiments were carried out in triplicate. *Blastocystis* sp. cells were enumerated based on morphology (vacuolar, granular, and amoeboid forms) for 10 days using a haemocytometer chamber (Improved Neubauer, Hausser Scientific, PA, USA) with 0.4% trypan blue dye exclusion (Sigma-Aldrich, MO, USA). Generation time (GT) was calculated for 24 h as described elsewhere [22]. A total of 100 cells were randomly picked from each field to measure the cell diameter.

### FITC staining for surface carbohydrate quantification

All *Blastocystis* sp. isolates (SZ: *n* = 9, NS: *n* = 5) from Day 3 culture were subjected to surface carbohydrate quantification following the protocol from a previous study [11]. Cell sediments of day 3 were washed with PBS twice (900×*g*, 5 min) and then diluted to 50 µl containing 10^6^ million cells. A volume of 50 µl of FITC-lectin solution (2 mg/ml Concanavalin A, Sigma, MO, USA) was added to the diluted cells and incubated at 37 °C for 30 min. The mixture was centrifuged (900×*g*, 5 min) and washed twice with PBS. The pellet was reconstituted to a volume of 50 µl with PBS, and a volume of 10 µl was examine by epifluorescence microscopy (Leitz, Wetzlar, Germany) at × 400 magnification. One hundred cells were randomly selected for visual intensity of fluorescence using the scale of brightness (0, 1 +, 2 +, 3 +, where 0 indicates no fluorescence and 3 + indicates highest fluorescence).

### Extraction of solubilised antigens from *Blastocystis* sp.

Purified *Blastocystis* sp. cells with negligible bacterial contamination were achieved using Ficoll-Paque density gradient centrifugation, and the solubilised antigens were extracted using freeze-thaw cycles as described in previous studies [37, 38]. The cell pellet was subjected to 30 freeze-thaw cycles using liquid nitrogen (1 cycle consisted of 1 min submerged in liquid nitrogen followed by 1 min in water bath at 37 °C). The lysate was then left at 4 °C overnight, followed by filter sterilisation and protein quantification using Bradford Assay. Aliquoted solubilized antigens were stored at − 20 °C for future usage.

### Quantification of proteases activity

Protease activity of *Blastocystis* sp. antigens was investigated using Azocasein Colorimetric Assay (1.5 mg/ml) as reported previously [21, 39]. The concentration of the solubilised antigen was standardised to 0.1 mg/ml. The antigens were incubated with Dithiothreitol (DTT) (2 mM) (Sigma-Aldrich, MO, USA) at 37 °C for the activation of proteases for 10 min. One hundred microlitres of antigens was added to 100 µl of pre-heated azocasein solution and incubated at 37 °C for 1 h. The reaction was stopped with 300 µl ice-cold trichloroacetic acid (Sigma-Aldrich, MO, USA), followed by ice incubation (30 min) and centrifugation at 8000 × *g* for 5 min. The supernatant was then treated with 500 µl of 500 mM NaOH followed by an absorbance reading at 440 nm (Infinite 200 Pro Nanoquant, Tecan, Mannedorf, Switzerland). All experiments were carried out in triplicates. Cell lysates inactivated in boiling water for 15 min were used as negative control, whereas trypsin (0.1 mg/ml) (Corning, MO, USA) was used as a positive control.

### Inhibition assay to determine specific protease activity using protease inhibitor

Protease inhibition assay was carried out to investigate the type of the most prevalent protease present among *Blastocystis* sp. isolated from SZ group according to previous studies [39, 40]. The protease activity of cysteine, serine, aspartic, and metalloprotease was measured using optimised concentration of protease inhibitors E-64 (0.1 mM) (Sigma-Aldrich, MO, USA), phenylmethanesulfonylfluoride (PMSF) (1 mM) (Sigma-Aldrich), iodoacetamide (Sigma-Aldrich), and EDTA (metalloprotease inhibitor) (1 mM) (Sigma-Aldrich), respectively. A volume of 100 µl of antigens (0.1 mg/ml) with different protease inhibitors was incubated with azocasein (5 mg/ml) for 1 h at 37 °C. The resultant mixture was treated with trichloroacetic acid, ice incubation, and treatment of NaOH (500 mM) following the protocol from the previous section. Percentage of protease activity inhibition after the addition of different inhibitors was calculated using the formula:$$Percentage of protease inhibition =\frac{P-Pi}{C}*100$$ where *P*: protease activity of cell lysate without inhibitors, *Pi*: protease activity after addition specific inhibitor azocasein as a substrate.

### Drug resistance studies

Three-day-old culture of *Blastocystis* sp. isolates was used for this subsection. *Blastocystis* sp. isolates were standardized to a concentration of 1 × 10^5^ cells/ml in fresh Jones medium supplemented with 10% horse serum and range of metronidazole concentration (0.0001, 0.001, 0.01, 0.1, 1 mg/ml). The growth of the cells and the morphology (amoebic, vacuolar, and granular forms) were monitored and recorded daily for 10 days.

### Colon cell culture proliferation studies

Effect of solubilised antigens from *Blastocystis* sp. on colon cancer cells (HCT116) was investigated as reported previously [37]. HCT110 cells were grown and maintained in full medium RPMI with L-glutamine (Corning) and antibiotics and supplemented with 5% foetal bovine serum (FBS) (Gibco Laboratories, Life Technologies, NY, USA) [37, 41]. A total of 1000 cells were seeded into 100-μl growth medium in 96-well plates and incubated overnight at 37 °C in 5% CO_2_. The next day, solubilised antigens (final concentration: 0.01 µg/ml) from each isolate were introduced to the cells in each well. Cell proliferation was calculated by carrying out MTT assay after additional 48 h treatment [42] using the following formula, $\mathrm{Cell proliferation }\left(\mathrm{\%}\right)=\frac{x-c}{c} \times 100$ where x = average OD of the wells of HCT116 cells after the treatment of solubilised antigen; c = average OD of the wells of HCT116 cells after the treatment of PBS + cell lysate wash.

### Statistical analysis

All statistical analysis was carried out using IBM SPPS software (version 23, NY, USA) and Microsoft Excel 2019 (Redmond, WA, USA). The threshold for statistical significance was *P* < 0.05.

## Results

### *Blastocystis* prevalence in schizophrenic patients

A prevalence of 24% (12/50) was detected among SZ by either xenic culture method (XCD) with Jones medium or PCR. Individually, among SZ samples, both methods reported a sensitivity of 83% (10/12) with each method unable to detect two different isolates, respectively. On the other hand, the NS cohort group demonstrated a total prevalence of 5% for both XCD and PCR. While there were significant differences (Fisher’s exact test: *P* = 0.0004) in the prevalence of *Blastocystis* sp. infection between the SZ and NS, no association of age [Mann-Whitney *U* (17) = 25.500, *Z* = − 0.476, *P* = 0.646] and gender (Fisher’s exact test: *P* > 0.05) with *Blastocystis* sp. infection was observed during the statistical analysis (Additional file 1: Table S1). In our study, we did not observe any significant differences between sensitivity of XCD and PCR detection methods. Nevertheless, our results demonstrated that 100% sensitivity could be achieved when both methods were run in parallel.

All 12 SZ individuals that were positive for *Blastocystis* sp. did not exhibit or report any presence of gastrointestinal symptoms. On the other hand, all five NS individuals that were positive for *Blastocystis* sp. were isolated from symptomatic individuals with either non-specific gastrointestinal issues (abdominal pain, bloating, and nausea) or loose stool.

### Genotypic analysis of *Blastocystis* sp. isolates from SZ and NS

Based on gene analysis of small-subunit ribosomal RNA (*SSU*-rRNA), a total of 15 *Blastocystis* sp. isolates were molecularly characterised revealing the presence of ST1 (20%, 3/15), ST3 (66.7%, 10/15), and ST6 (13.3%, 2/15). Out of the ten SZ isolates, eight were of subtype 3 with the exception of SZ6 and SZ10, which were subtype 1. As for the NS isolates, two out of five were of ST3 and ST6 each with NS1 belonging to ST1. Chi-square test: *χ*^2^ = 2.179, df = 2, *P* = 0.36 indicated there was no significant association between the subtype frequency and the cohort group (SZ or NS). We detected a few of different *Blastocystis* subtype alleles in our samples. ST3 isolates with alleles 34 accounted 90% of the samples whereas one isolate was with allele 36. Interestingly, the intra-subtype variation of ST3 was only observed in the SZ cohort group. Both the ST6 isolates from the NS cohort group are of different alleles (122 and 139). No intra-subtype variation was observed among the ST1 isolates as all of the isolates had allele 4. All the sequences have been deposited into the GenBank (accession number: ON564757-ON564771).

### Growth profile

**Fig. 1:**
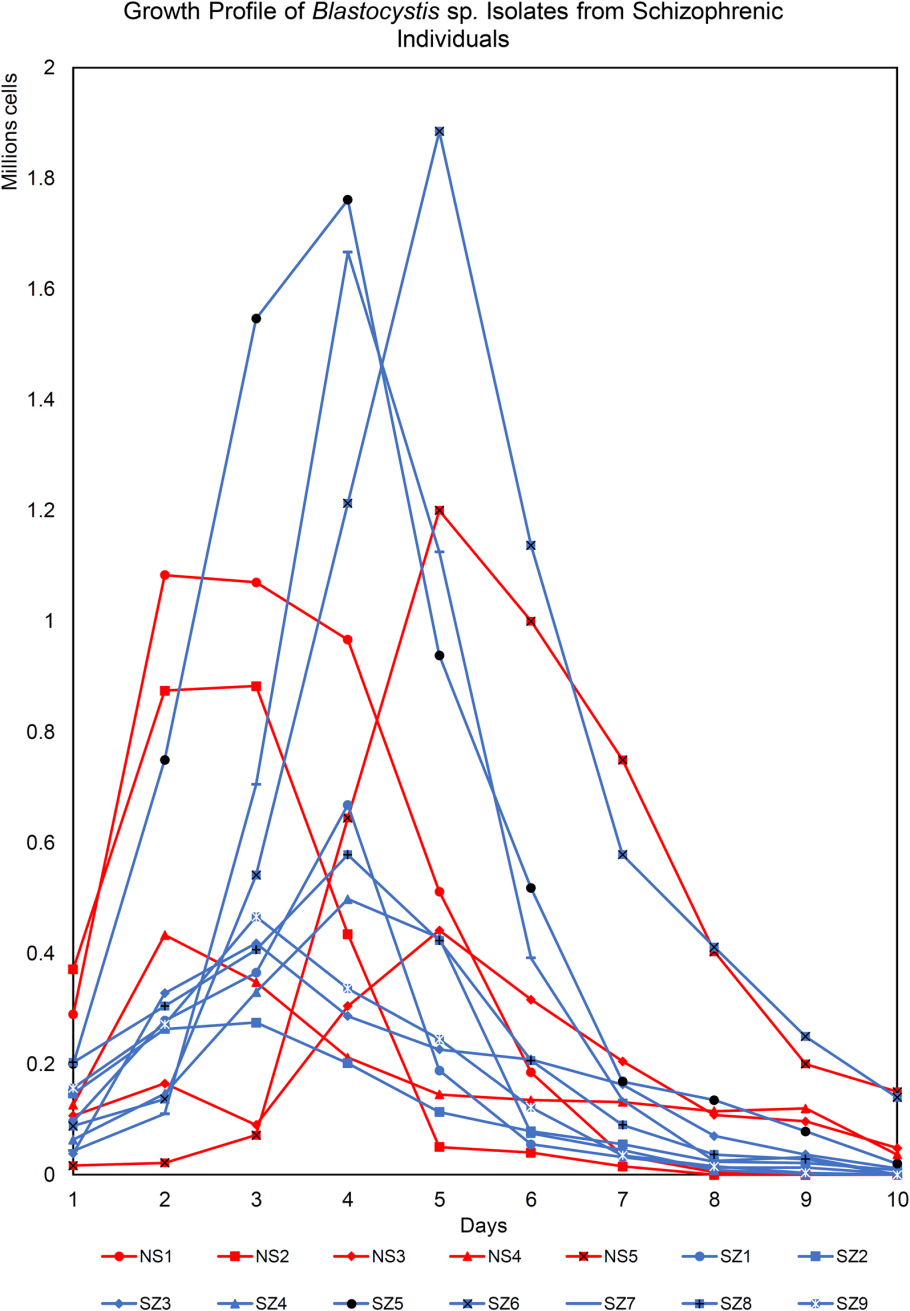
Growth profile of *Blastocystis* sp. isolates from schizophrenic individuals. *Blastocystis* sp. isolated from schizophrenic (blue line) and non-schizophrenic (red line) individuals were grown in Jones medium supplemented with 10% horse serum for a period of 10 days

**Fig. 2:**
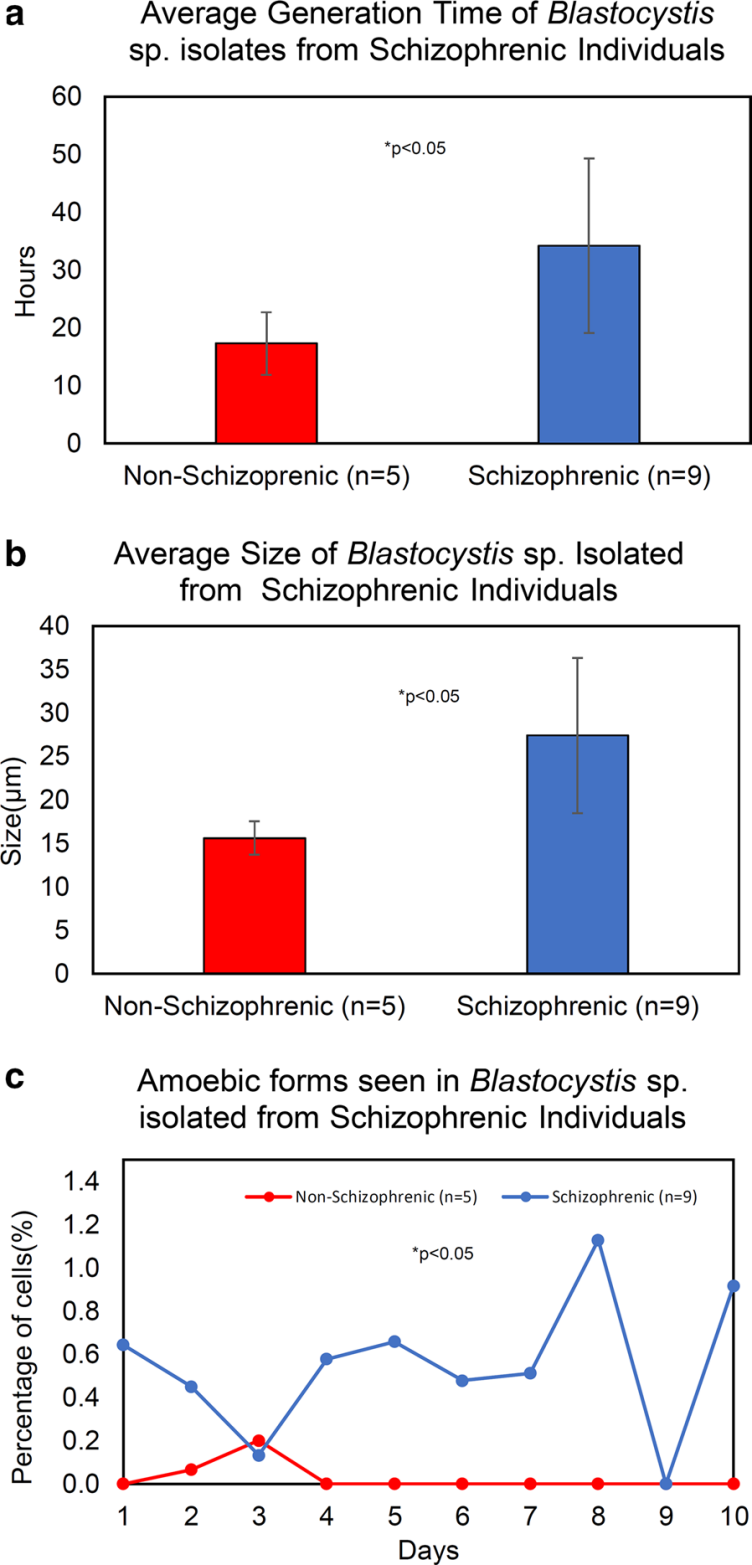
**a** Average generation time of *Blastocystis* sp. **b** Average size of *Blastocystis* sp. cells. **c** The number of amoebic forms observed across a period of 10 days. Each point represents an average of all amoebic forms in respective cohort groups. Red denotes non-schizophrenic and blue indicates schizophrenic isolates. *P < 0.05 for respective statistical analysis

A total of 14 *Blastocystis* sp. isolates (SZ: *n* = 9, NS: *n* = 5) were studied for the growth profile and morphometry for 10 days (Figs. 1, 2 and Additional file 2: Table S2). Generally, NS isolates consistently demonstrated growth profile with high growth peaks whereas SZ isolates demonstrated a wider range of growth peaks with most having low growth peaks. One third of SZ isolates (SZ5, SZ6, and SZ7) recorded the highest peak out of all isolates put together, 1.8, 1.9, and 1.7 × 10^6^ cells/ml. NS isolates grew rapidly when compared to SZ isolates [Mann-Whitney *U* (14) = 4, Z = − 2.467, *p* = 0.014] with an average generation time of 17.31 ± 5.4 h and have smaller cells with consistent diameter (3.3–34 µm). On the other hand, SZ isolates have a longer generation time of 34.9 ± 15.1 h. Generally, the diameters of the cells from SZ isolates were significantly greater [independent *t* test: *t* (12) = 2.578, *P* = 0.024] with the largest diameter around 140 µm (Additional file 3: Fig. S1). More amoebic forms [Mann-Whitney *U* (10) = 7, *Z* = − 3.408, *P* = 0.001) are also observed among SZ isolates after Day 3 when compared to NS isolates.

### Lectin staining

**Fig. 3:**
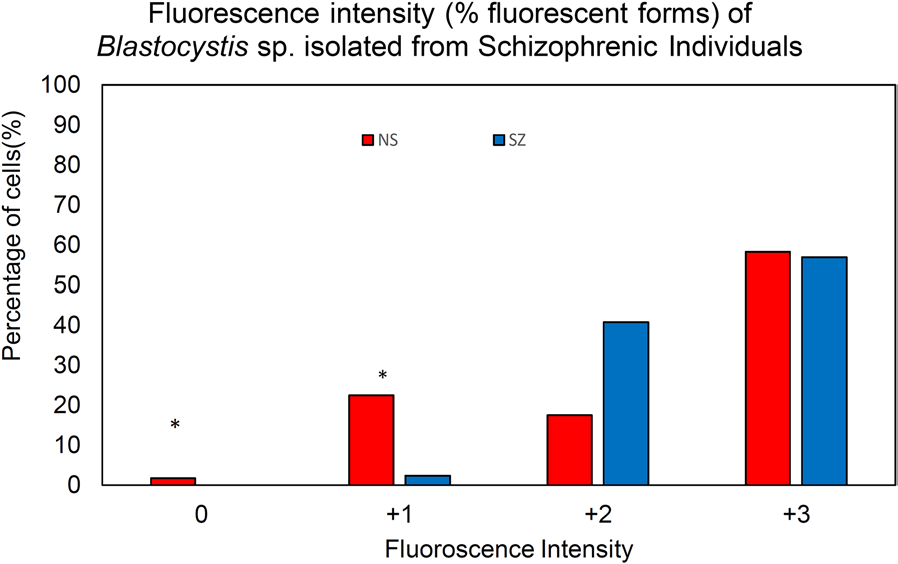
Fluorescence intensity of *Blastocystis* sp. isolated from schizophrenic individuals. AFU 0: no fluorescence, AFU 1: + weak intensity, AFU 2 +: medium strong AFU 3 +: strong intensity (percentage of reactive forms). *p < 0.05 in independent *t*-test for comparison of means of fluorescence intensity

Both SZ and NS isolates predominantly demonstrated high fluorescence intensity (AFU2 ± FU3 +) towards FITC-ConA (2 mg/ml) (Fig. 3 and Additional file 4: Table S3). However, NS isolates demonstrated a broader range of fluorescence intensity with most NS isolates (NS1–NS4) having slightly greater binding affinity than all SZ isolates. While no weak binding (AFU 0) was observed among SZ isolates, significant low lectin binding was observed among NS isolates, AFU 0 [Mann-Whitney U(14) = 9, *Z* = − 2.51, *P* = 0.012]) and AFU + 1 [Mann-Whitney *U*(14) = 6.5, *Z* = − 2.182, *P* = 0.029]. Agglutination of cells was observed in two NS isolates (NS3 and NS4) and two SZ isolates (SZ5 and SZ9) (Additional file 5: Fig. S2).

### Drug resistance

**Fig. 4:**
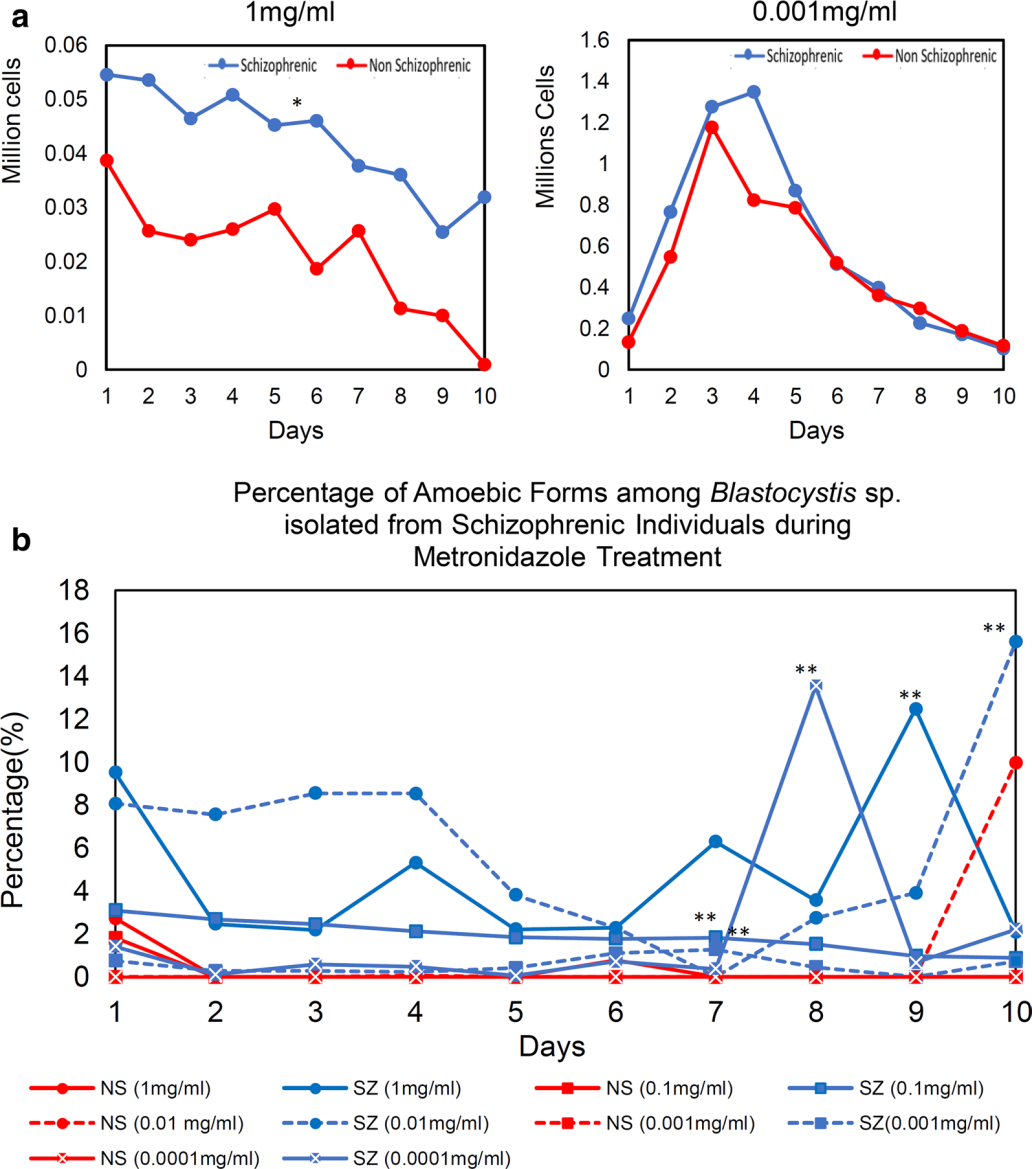
**a** Antibiotic resistance of *Blastocystis* sp. against metronidazole treatment (1 mg/ml and 0.001 mg/ml). **b** Percentage of amoebic form among *Blastocystis* sp. isolated from schizophrenic individuals. Each data point represents average of number of cells resistant towards metronidazole in respective cohort groups. *P < 0.05 in independent *t*-test for comparison of means of peak of cells. **P < 0.05 in Mann-Whitney U test for comparison of means of peak percentage amoebic forms

All *Blastocystis* sp. isolates were treated with metronidazole at two different concentrations (1 mg/ml or 0.0001 mg/ml, Fig. 4). Overall, SZ isolates showed stronger resistance towards antibiotics as a higher cell count was observed even at higher metronidazole concentration of 1 mg/ml [independent *t* test: *t* (10) = 4.691, *P* = 0.0002] (Fig. 4a). In addition, the SZ isolates were able to sustain and proliferate beyond day 10 in all metronidazole treatments whereas most NS isolates die nearing day 10. At 0.001 mg/ml), both SZ and NS demonstrated no significant difference [independent *t* test: *t* (10) = 4.31, *P* = 0.345] against metronidazole resistance with maximum growth and decrease in average generation time in medium with 0.001 mg/ml metronidazole when compared to the control without antibiotic treatment. Treatment of metronidazole significantly increased the amoebic forms in SZ isolates. The percentage of amoebic form was more observed among SZ isolates compared to NS isolates (Fig. 4b). Mann-Whitney (*U*) test showed there was significant difference of the percentage of amoebic forms observed between SZ and NS after the treatment of metronidazole at both concentrations (1 mg/ml and 0.0001 mg/ml). There was an increment of 2–16% of amoebic forms during the treatment with metronidazole. The highest elevation of amoebic forms was observed during the treatment with 0.01 mg/ml metronidazole in SZ isolates [Mann-Whitney *U* (19) = 5, *Z* = − 3.514, *P* = 0.0004]. In addition, more granular forms were observed in both isolates after the metronidazole treatment.

### Protease Activity of *Blastocystis* sp. isolated from SZ and cell proliferation of HCT116 cell line

**Fig. 5:**
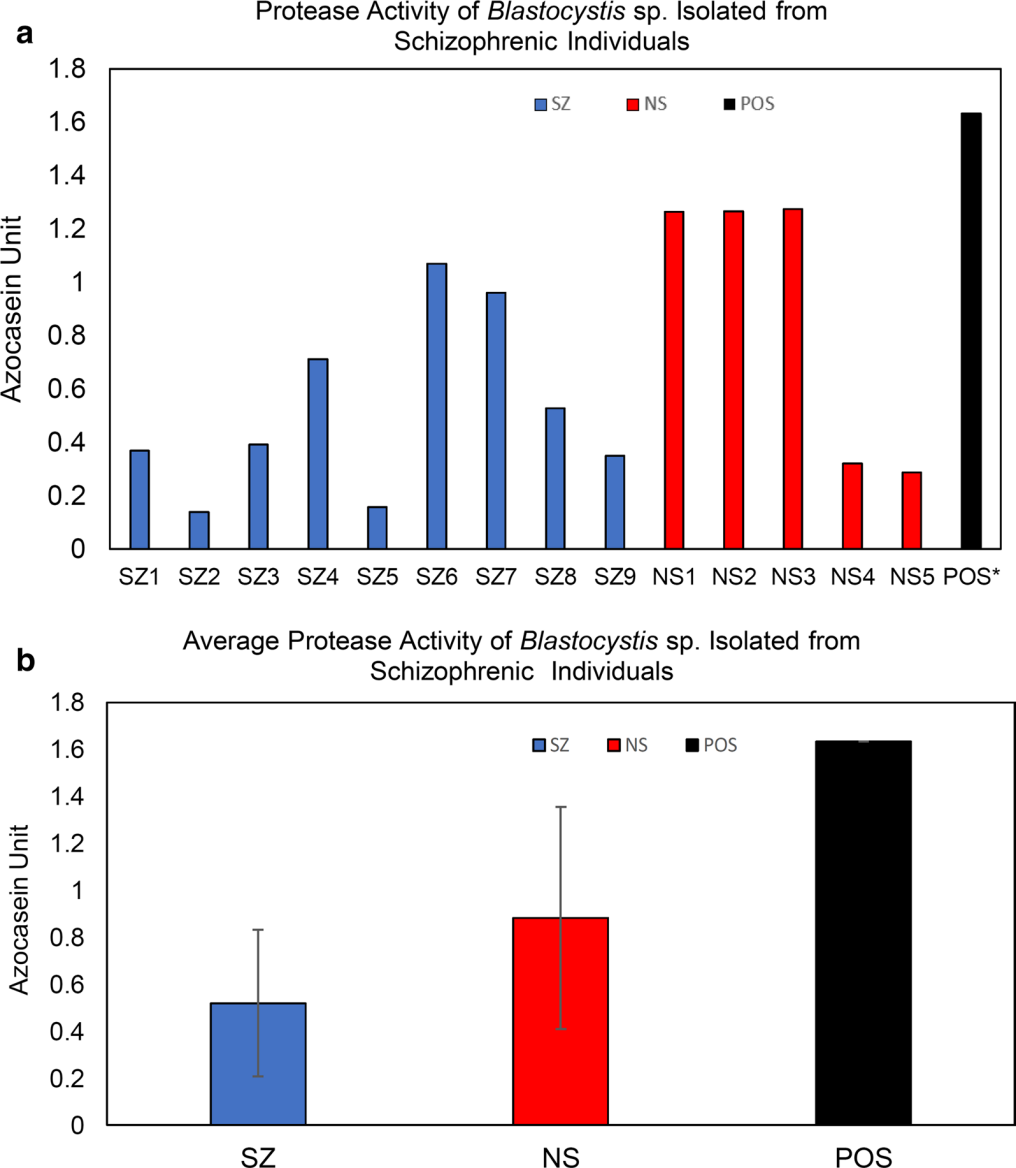
**a** Protease activity and **b** Average protease activity of *Blastocystis* sp. isolates from schizophrenic individuals. The concentration of solubilised antigens was standardized to 0.1 mg/ml and trypsin (0.1 mg/ml) was used as a positive control. *SZ* Schizophrenic individuals, *NS* non-schizophrenic individuals

**Fig. 6:**
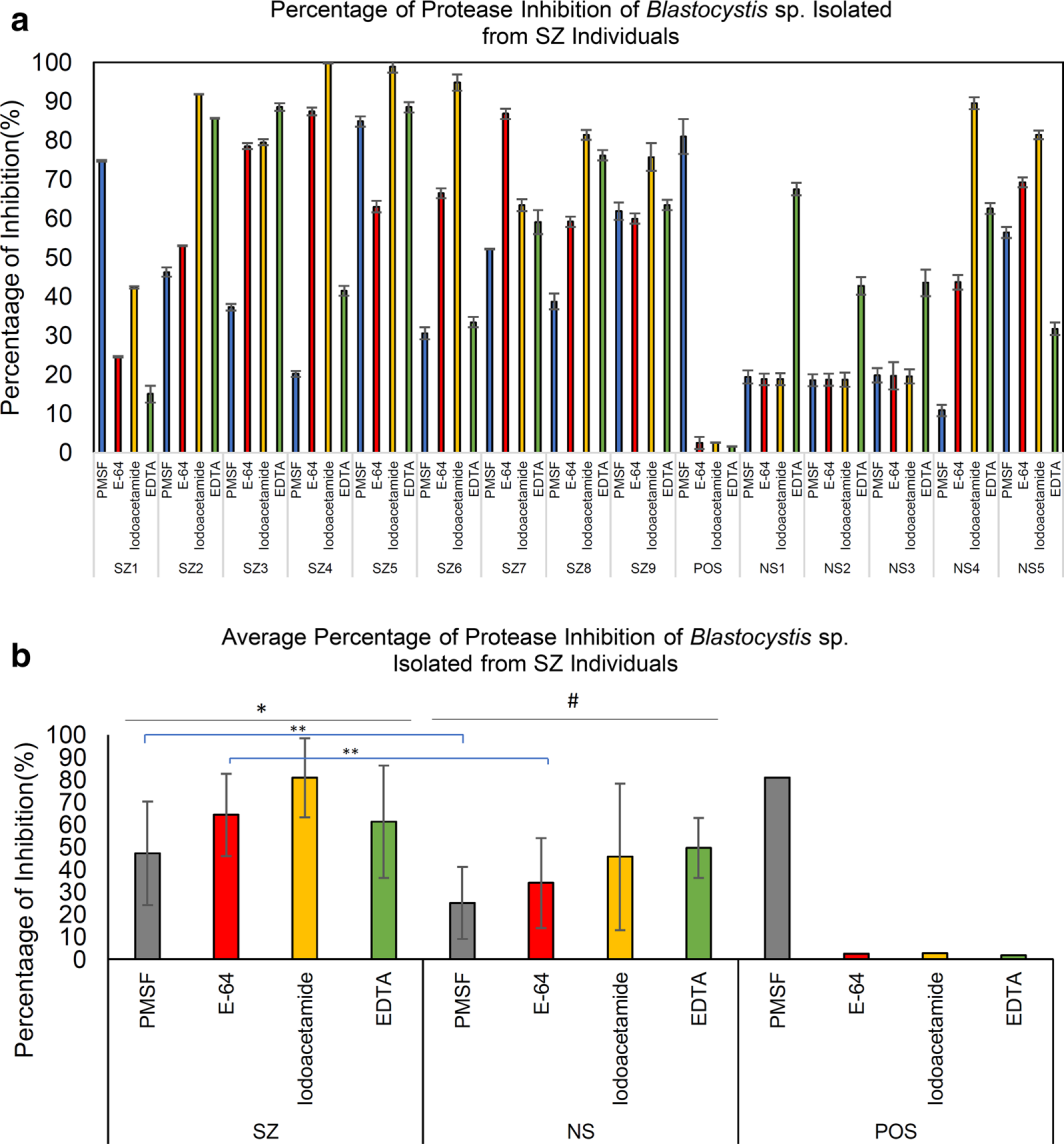
**a** Percentage of protease inhibition and **b** average of protease inhibition of *Blastocystis* sp. isolated from schizophrenic individuals. The concentration of solubilised antigens was standardized to 0.1 mg/ml whereas trypsin (0.1 mg/ml) was used as positive control. Values were obtained from an average of three independent experiments with triplicates in each sample. *PMSF* Phenylmethylsulfonyl fluoride, *EDTA* ethylenediaminetetraacetic acid, *SZ* schizophrenic individuals; *NS* non-schizophrenic individuals. Horizontal black lines: *P < 0.05, ^#^*P* > 0.05 in ANOVA analysis for mean comparison of percentage of protease activity inhibition within a cohort group. Horizontal blue lines: **P < 0.05 in independent *t*-test for comparison for means of percentage of protease inhibition between SZ and NS

Protease activity using the azocasein assay was investigated among *Blastocystis* sp. from SZ individuals. In general, the total protease activity of *Blastocystis* sp. from SZ was lower than of isolates from NS (Fig. 5). A total of seven out of nine SZ isolates demonstrated high protease activity whereas around two thirds of NS isolates had high protease activity. Mann-Whitney *U*-test *U* (14) = 14.00, *Z* = − 1.133, *P* = 0.257, showed no significant difference of the total protease activity between SZ and NS. Protease inhibition assay indicated there was significant [ANOVA: *F* (3.35) = 3.196, *P* = 0.037] inhibition of protease activity among SZ isolates in particular by cysteine inhibitors such as E-64 and iodoacetamide suggesting predominance of cysteine protease (Fig. 6a). In contrast, high inhibition by EDTA in NS isolates suggests predominance of metalloprotease. Independent *t*-test analysis was carried out to investigate the significance difference of protease inhibition between SZ and NS cohort groups. There was significant difference of protease inhibition by PMSF [*t*-test: *t* (12) = 2.201, *P* = 0.044) and E-64 [*t*-test: *t* (12) = 2.546, *P* = 0. 037] between the two cohort groups. This suggests significantly higher activity of cysteine and serine protease in *Blastocystis* sp. isolated from SZ individuals.

**Fig. 7:**
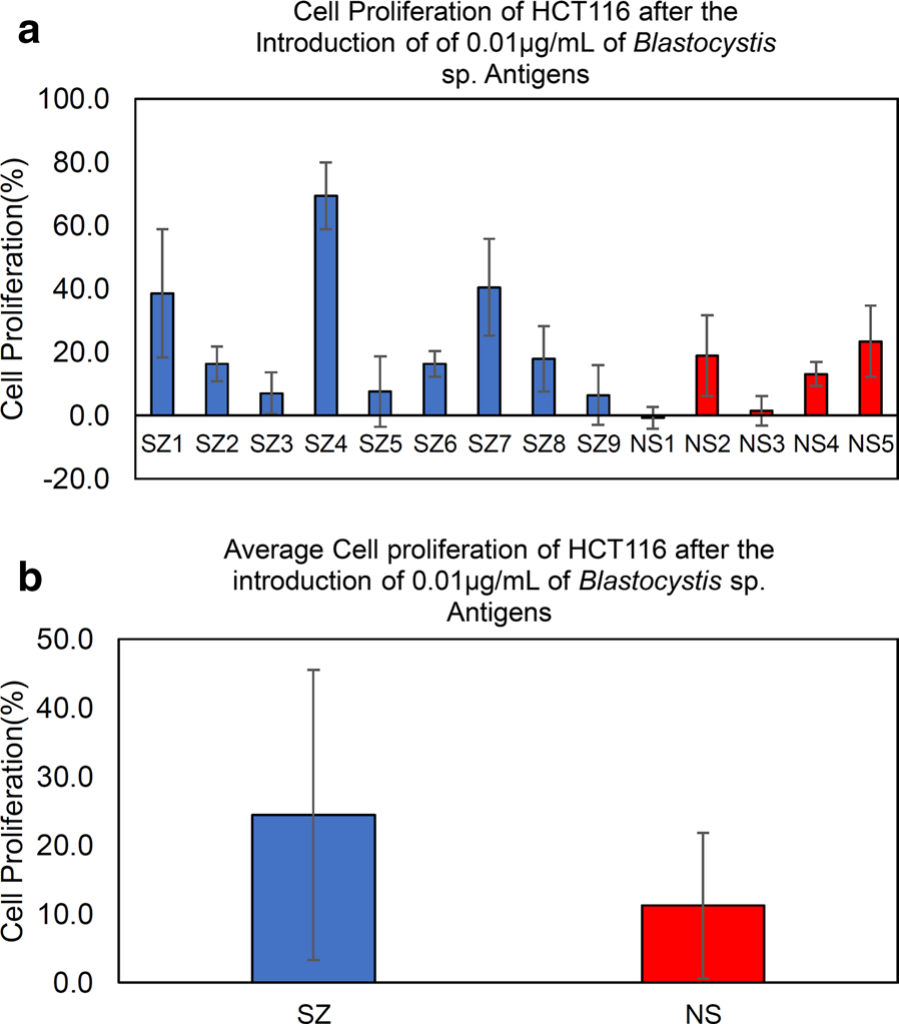
**a** Cell proliferation. **b** Average cell proliferation of HCT116 after the treatment of solubilised antigens of *Blastocystis* sp. from SZ individuals. The final concentration of the solubilised antigens was standardized to 0.01 µg/ml. MTT assay was carried out to measure the cell proliferation. *SZ* Schizophrenic individuals, *NS* non-schizophrenic individuals

**Fig. 8:**
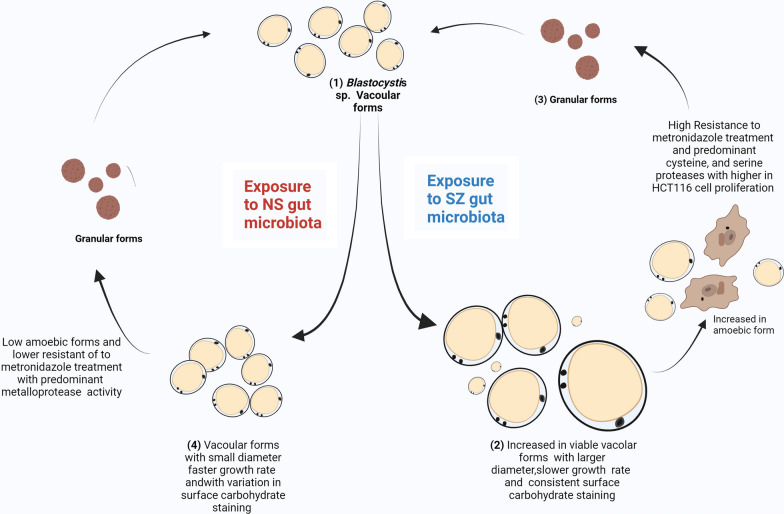
Schematic diagram depicting the major findings in the current study. One of the critical findings in this study is that the life cycle and the morphology of *Blastocystis* sp. are host dependent and undergo alteration because of the host microenvironment such as the gut microbiota. (1) Exposure of the SZ gut microbiota causes an alteration of in phenotypic characteristics of *Blastocystis* sp. such as increased parasite diameter up to 140 µM and longer average generation time of 34.9 ± 15.06 h. SZ isolates consistently showed a medium to high lectin staining indicating rough surface morphology. (2) There was also a significant increase in amoebic forms among SZ isolates, which resulted in higher resistance of metronidazole treatment with predominant cysteine and serine protease activity. The SZ isolates also demonstrated higher average cell proliferation of HCT116 cell line. (3) The resultant granular forms are more robust and produces more viable vacuolar forms of the parasite [100]. (4) The NS isolates exhibited higher growth rate (17.31 ± 5.4) with consistently smaller diameter (34 µM). NS isolates demonstrated variation in surface lectin staining (predominantly high with smaller percentage showing little to no fluorescence) and low amoebic forms. NS isolates also showed lower resistance towards metronidazole treatment with predominant metalloprotease activity and low proliferation of HCT166 cell line. SZ denotes individuals with schizophrenia whereas NS denotes non-schizophrenic individuals

We attempted to investigate the effect on the cell proliferation of the HCT116 cancer cell line after the treatment of solubilised antigen (0.01 µg/ml) from *Blastocystis* sp. Our results (Figs. 7 and 8) demonstrated the cancer cell proliferation in average was elevated after the introduction of solubilised antigens from both cohort groups especially among SZ isolates.

## Discussion

The gut microbiome is a consortium of trillions of microorganisms including bacteria, fungi, and protozoa such as *Blastocystis* sp. [43]. Colonisation by *Blastocystis* sp. has been associated to multiple gastrointestinal issues such as IBS [44, 45], ulcerative colitis [46], and colorectal cancer (CRC) [41, 47]. Studies in the past have reported phenotypic variation of *Blastocystis* sp. when isolated from different sources [22]. This has been attributed to variation in gut microbiome. Protozoa that were isolated from different gut microbiomes displays different phenotypic and genotypic traits and assessment of these variations is crucial in understanding patterns associated to pathogenic potential of a microorganism [48]. In this study, for the first time, we investigated the characteristics of *Blastocystis* sp. isolated from SZ and NS individuals that reportedly had significant different gut microbial composition [49, 50]. A study investigating the gut microbiome of high-risk SZ individuals from China reported an increase of relative abundance of various taxa in particular among the order of *Bacteroidales*, *Clostridiales*, and *Lactobacillales* [51]. Another study compromising SZ patients under antipsychotic medications concurred with the findings and reported an increase of these taxa among SZ [52]. In addition, gut microbiome studies involving drug-naïve SZ patients before and after antipsychotic medication treatment reported that the medication caused further intestinal dysbiosis and can be used as a measure for severity of SZ [53, 54]. While the potential of antipsychotic medication-induced gut microbiota alteration in aiding the colonisation of *Blastocystis* sp. is still unknown, it cannot be ruled out entirely. It is possible that gut microbiota of treated SZ may have aided the colonisation of *Blastocystis* sp. and contributed to the morphological differences seen.

*Blastocystis* sp. infections among the mentally ill have been reported previously at a prevalence of 4–55% in Iranian individuals from caring homes and rehabilitation centres [47, 55]. Our study reports a *Blastocystis* sp. prevalence of 24% among chronic SZ patients consistent with the growing literature. A study from Iran reported the prevalence of ST3 among SZ patients [31]. Similarly, our results indicated ST3 to be the most prevalent subtype among both cohort groups especially in SZ isolates (66.7%). This difference could be explained by variation of subtype distribution according geographic locations where ST3 is found most predominantly in Southeast Asia [56] and specifically in Malaysia [3]. We have also observed ST6 (closely related to ST6 reference sequences from partridge and quail) in the NS samples. Infection with ST6 is rare among humans and could possibly reflect cases of zoonotic transmission as reported previously [57, 58].

In this study, the SZ individuals with *Blastocystis* sp. infection did not report or showcase any obvious gastrointestinal symptoms except for frequent constipation, which is common because of the consumption of antipsychotic drugs [59]. Individuals with schizophrenia are frequently reported to have reduced sensitivity to pain [60] and the exact reasons are likely multifactorial because of the complex aetiology of SZ. Developments of pre-frontal cortex, mediodorsal thalamus impairment, and alteration of dopamine or neurotransmitters were among the few factors that have been cited to play a role in reduced pain sensitivity [61]. Therefore, we presume that these factors could have resulted in impairment of sensing gastrointestinal pain and hence no symptoms were reported by SZ individuals. Moreover, post-autopsy studies on SZ individuals with higher rates of IBS, colitis, gastritis, and enteritis [62, 63] suggest the presence of gastroenterological-related damages that can go undetected because of reduced pain sensitivity. There are possibilities that symptoms such as abdominal pain were not detected as a result of reduced pain sensitivity. On the other hand, multiple studies have demonstrated various enteric protozoan infections without symptomatic disease [64, 65]. This may also possibly explain the lack of any gastrointestinal symptoms among SZ individuals.

In this investigation, six out of nine *Blastocystis* sp. isolated from SZ individuals demonstrated slower growth rates and greater diameters, which is consistent with *Blastocystis* sp. isolated from symptomatic and IBS patients [18, 34]. We noticed that the remaining three SZ isolates demonstrated significantly high growth peaks with smaller diameters as commonly seen in asymptomatic isolates [66]. This suggests that the growth profile of SZ isolates demonstrates inconsistent growth peaks as seen in both symptomatic and asymptomatic isolates. The current findings are contradictory to other studies where *Blastocystis* sp. that are isolated from hosts with the same affliction tend to demonstrate a similar pattern of phenotypic characteristics [11, 22]. More studies are warranted to further shed light on the discrepancy in growth profile among SZ isolates. Nevertheless, SZ individuals being prone to developing IBS [67] raises the possibility that some of our subjects (SZ individuals with *Blastocystis* sp. infection) may also have undiagnosed IBS, therefore explaining the morphological similarity between most of the SZ *Blastocystis* sp. isolates and IBS isolates in our previous study [22].

Con-A FITC binding analysis revealed that most of the isolates from both cohort groups showed high to medium fluorescence intensity but greater consistency was seen in SZ isolates. This finding is consistent with previous studies reporting the abundance of surface carbohydrate on *Blastocystis* sp. [15, 68]. Surface glycoproteins such as lectin are crucial for the adherence to the intestinal lining [69] and interaction with gut bacteria [68] and have been reported to aid in the pathogenicity of microorganisms [70, 71]. Besides, Con-A FITC staining has been previously used to distinguish symptomatic (high fluorescence) from asymptomatic isolates (low fluorescence) [11, 18]. High and consistent fluorescence among SZ isolates indicates a rougher surface topology similar to symptomatic isolates such as *Blastocystis* sp. from IBS patients [22]. On the other hand, a wide variation of fluorescence among NS isolates implies NS5 (low fluorescence) behaves similarly to asymptomatic isolates whereas the remainder (high fluorescence) behaves similarly to symptomatic. The variation observed among NS isolates could be host dependent.

Metronidazole resistance in *Blastocystis* sp. has become increasingly prevalent [66]. In our study, cell growth of both NS and SZ isolates increased drastically at 0.001 mg/ml metronidazole treatment suggesting resistance forms as reported previously [66]. This shows that metronidazole should be carefully administered as an inappropriate concentration of the drug may result in resistance [72]. We also observed that *Blastocystis* sp. isolated from SZ patients has greater ability to withstand high metronidazole concentrations in vitro. This indicates greater robustness in *Blastocystis* sp. cells when isolated from SZ individuals and it could be because these isolates have been pre-exposed to various medications and anti-psychotic drugs usually prescribed for SZ. Introduction of drugs alters the gut microbiome to withstand harsher environments [73]. Therefore, *Blastocystis* sp. isolated from this environment may have greater vigour in harsh environments as well. Studies have proven that IBS patients demonstrated 60% metronidazole resistance [74, 75] suggesting symptomatic isolates to be more resistant to metronidazole treatment. These findings once again indicate SZ isolates behave similarly to symptomatic isolates especially from IBS individuals. The viability of SZ isolates at a higher concentration of metronidazole indicates greater robustness and suggests increased risk of transmission and infectivity of *Blastocystis* sp. when isolated from these individuals.

Amoebic forms are often associated to pathogenic potential as they are mostly found in symptomatic isolates [21, 76], possess a sticky surface [19], and contain more proteases [39]. Higher prevalence of amoebic form has been reported in *Blastocystis* sp. isolated from symptomatic and patients with IBS [19, 76]. In the current study, amoebic morphological forms were more pronounced in SZ isolates especially during metronidazole treatment. Increase in amoebic forms among SZ isolates raises the potential of pathogenicity of these isolates. These parasitic cells, which also demonstrated a higher percentage of medium to high surface carbohydrate, suggest enhanced interaction with gut bacteria. Past studies have reported the ability of *Blastocystis* sp. to engulf gut bacteria [77] and influence microbial composition [78, 79]. A recent study has postulated that *Blastocystis* sp. is predacious against highly abundant bacterial taxa in the gut of asymptomatic individuals [80]. A detailed metagenomic analysis would shed light if a similar effect is seen in *Blastocystis* sp. infection among SZ individuals. Moreover, recent research demonstrated evidence on the use of probiotics/prebiotics to positively influence SZ patients [81]. Whether *Blastocystis* sp. could interfere such interventions in SZ patients is also obscure.

Our findings reported the total average protease activity of SZ isolates was lower than for NS isolates despite demonstrating higher amoebic forms. This is in contrast to a previous study linking amoebic forms and proteases [21]. Possible explanation to this observation could be due to the fact that in our study we saw much lower amoebic forms among SZ isolates. In vitro and in vivo studies have previously established that induction of stress enhanced the infectivity in *Blastocystis* sp. such as an upsurge in cyst counts, suppression of immune response, and imbalances of stress-mediated augmentation [82]. Similarly, the increase in amoebic forms could also be another response to environmental stress such as antipsychotic medications in order to survive harsh condition. Our findings further corroborate previous sentiments that the life cycle of *Blastocystis* sp. and predominance of certain forms (vacuolar, granular or amoebic) are host dependent and are greatly affected by the host gut microbial environment.

Proteases from *Blastocystis* sp. reportedly play various roles such as degrading mucosal IgA [83], disrupting tight junctions leading to increased intestinal permeability [84] and increased inflammation via IL8 mediation [13]. Cysteine protease is the predominant type frequently reported among *Blastocystis* sp. [85–87]. In this study, we noticed high presence of cysteine and serine protease activity among SZ isolates due to greater inhibition by iodoacetamide. These proteases aid the survival of protozoa and are crucial in degradation of gut protein such immunoglobulin, haemoglobin, and intestinal membrane and wall [88, 89]. Cysteine proteases acts as virulent factors as was seen predominantly in pathogenic strains of *Entamoeba histolytica* [90]. Although protease as a virulent factor is not established in *Blastocystis* sp., high levels are seen together with other phenotypic characteristics, elevating the possibilities of pathogenic-like qualities in SZ isolates. In addition, higher presence of metalloproteases was also observed in *Blastocystis* sp., specifically among NS isolates. Although the difference was not statistically significant, the observation indicates that predominance of a specific protease can be influenced by the source of isolation. Metalloproteases are reported to be crucial in modulating host immune response as well as pathogenesis in parasitic disease [91]. Whole-genome sequencing of *Blastocystis* revealed that the protozoan is also capable of producing metalloprotease apart from the 20 types of cysteine proteases [86, 92]. Metalloproteases have also been identified to be predominantly expressed among symptomatic *Blastocystis* sp. isolates from individuals with IBS [93].

We noticed that cancer cell proliferation was generally enhanced after the treatment with solubilised antigens from *Blastocystis* sp. cells. In our study solubilised antigens from *Blastocystis* sp. isolated from SZ individuals promoted the cell proliferation two-fold higher. Generally, human-derived cysteine proteases and serine proteases have been linked to cell proliferation of cancer cells [94]. Coexpression of host-derived serine proteases such as trypsin and metalloproteases facilitates invasion and metastasis of colorectal cancer [95]. Predominance of cysteine and serine proteases among the SZ *Blastocystis* sp. isolates was observed in this study and opportunistic infection of *Blastocystis* sp. in CRC was recently demonstrated [96]. Whether the protozoan-derived proteases could contribute to such cancer pathogenesis is unknown. Currently, it is only understood that *Blastocystis* sp. cysteine protease stimulates secretion of IL-8 indicating gut inflammation [97, 98]. However, more evidence on molecular mechanism of *Blastocystis* sp. protease activity in inflammation and immune regulation is required to associate this organism to cancer pathogenesis.

While the present study highlights unique traits of *Blastocystis* sp. when isolated from SZ individuals, it also implies the risks when the organism is transmitted to non-infected individuals as they possess characteristics (heightened amoebic forms and metronidazole resistance) suggesting greater infectivity and transmissibility. A study revealed that healthcare of SZ outpatients is often neglected and gastrointestinal-related problems need dire attention [99]. This study shows the need for the diagnosis and treatment of *Blastocystis* sp. in routine health screening procedures in SZ patients as uncertain tendencies of this organism cause symptomatic infection when it turns opportunistic, which should not be neglected. Furthermore, many SZ patients in Malaysia reside in mental institutions and nursing homes, where they live in close quarters with many other residents. These living conditions can easily facilitate the spread of *Blastocystis* sp. to a large number of other psychiatric patients if unchecked.

## Conclusion

The present study reports variation in phenotypic characteristics among *Blastocystis* sp. isolated from SZ patients. Our findings indicate *Blastocystis* sp. isolated from SZ individuals possess characteristics that are similar to symptomatic isolates with a higher number of amoebic forms and stronger resistance towards metronidazole treatment. These findings add to the growing literature showing that the physical properties of microorganisms such as *Blastocystis* sp. are greatly affected and altered by the gut micro-environment and the life cycle of the intestinal colonizer is host dependent. Our findings also highlight that it may be difficult to eradicate *Blastocystis* sp. isolates from individuals with harsh gut microbiomes such as those with SZ with metronidazole treatment. This indicates individuals with chronic SZ have a higher risk of transmission within a community especially in a caring home or health facilities. In addition, enhanced pathogenic-like qualities among SZ isolates increase the risk of aggravating cancer growth among SZ individuals with pre-existing colon/rectal cancer.

## Supplementary Information

**Additional file 1: Table S1. **Prevalence of *Blastocystis *sp. in SZ and NS group.

**Additional file 2: Table S2**. Generation time, number of generations, and size of *Blastocystis* sp. isolated from schizophrenic patients (SZ1-SZ9) and non-schizophrenic individuals (NS1-NS5).

**Additional file 3: Fig. S1.** Size variation observed among *Blastocystis* sp. isolated from **a** non-schizophrenic and **b** schizophrenic individuals. **c**, **d** Amoebic forms observed among *Blastocystis *sp. isolated from schizophrenic patient (SZ6). Arrows indicates amoebic forms, and all images are viewed at 400× magnification.

**Additional file 4: Table S3.** Intensity of fluorescence and percentage of fluorescent forms of* Blastocystis *sp. labelled with FITC-labelled lectins.

**Additional file 5: Fig. S2.** Microscopic view of *Blastocystis *sp. stained with FITC-labelled Concanavalin A. **a** Schizophrenic isolates with AFU 2+ fluorescence (400×) and **b** schizophrenic isolates with AFU+3 fluorescence (100×). **c** Non-schizophrenic isolates with AFU 2+ fluorescence (400×) and **d** non-schizophrenic isolates with AFU +1 and AFU 0 fluorescence (400×). AFU 0: no fluorescence, AFU 1: + weak intensity, AFU 2+: medium strong, AFU 3+: strong intensity (percentage of reactive forms).

## Data Availability

The authors confirm that the data supporting the findings of this study are available within the article. Raw data that support the findings of this study are available from the corresponding author, upon reasonable request.
