# Supplementary material for: Higher amoebic and metronidazole resistant forms of Blastocystis sp. seen in schizophrenic patients
Source: Parasit Vectors. 2022 Sep 5;15:313. doi: 10.1186/s13071-022-05418-0 (PMC9446727; doi:10.1186/s13071-022-05418-0)
Supplement: Supplementary file 1 — Additional file 1: Table S1. Prevalence of Blastocystis sp. in SZ and NS group. [file 13071_2022_5418_MOESM1_ESM.docx]

**Table S1: Prevalence of *Blastocystis* sp. in SZ and NS group**

|  | *Blastocystis* sp*.* Positive | | |
| --- | --- | --- | --- |
| Parameters | SZ (*n* = 12) | NS (*n* = 5) | *P* value |
| Age | 58 | 60.8 |  |
| Median age (years) | 66 | 61 | 0.646^a^ |
| Sex |  |  |  |
| Male | 8 (66.66%) | 3 (60%) | 0.61^b^ |
| Female | 4 (33.33%) | 2 (40%) |  |
| *Blastocystis sp*. Positive infection |  |  |  |
| Present | 12 (24%) | 5 (5%) | 0.0004^b^ |
| Absent | 48 (76%) | 95 (95%) |  |

a Mann–Whitney U test

b Fisher’s exact test
