# Supplementary material for: Higher amoebic and metronidazole resistant forms of Blastocystis sp. seen in schizophrenic patients
Source: Parasit Vectors. 2022 Sep 5;15:313. doi: 10.1186/s13071-022-05418-0 (PMC9446727; doi:10.1186/s13071-022-05418-0)
Supplement: Supplementary file 2 — Additional file 2: Table S2. Generation time, number of generations, and size of Blastocystis sp. isolated from schizophrenic patients (SZ1-SZ9) and non-schizophrenic individuals (NS1-NS5). [file 13071_2022_5418_MOESM2_ESM.docx]

**Table S2**: Generation time, number of generation and size of *Blastocystis* sp. isolated from schizophrenic patients (SZ1-SZ9) and non-schizophrenic individuals (NS1-NS5).

| Isolates | Period of Maximum Growth | Generation Time (h) | Number of Generations | Size (µm) |
| --- | --- | --- | --- | --- |
| NS1 | Day 1–2 | 12.7 | 1.9 | 16.8± 3.3 |
| NS2 | Day 1–2 | 19.6 | 1.2 | 14.6±1.2 |
| NS3 | Day 3–4 | 13.7 | 1.7 | 15.2±7.9 |
| NS4 | Day 1–2 | 13.6 | 1.8 | 19.9±8.8 |
| NS5 | Day 4–5 | 27.0 | 0.9 | 12.8±6.6 |
| Mean ± SD |  | 17.3 ±5.4 |  | 15.6±1.9 |
| SZ1 | Day 3–4 | 27.7 | 0.9 | 28.6±21.4 |
| SZ2 | Day 2–3 | 28.6 | 0.8 | 38.2±32.1 |
| SZ3 | Day 2–3 | 69.1 | 0.3 | 23.9±15.2 |
| SZ4 | Day 2–3 | 40.6 | 0.6 | 25.8±23.7 |
| SZ5 | Day2–3 | 23.1 | 1.0 | 13.3±4.3 |
| SZ6 | Day 3–4 | 20.8 | 1.2 | 17.4±7.8 |
| SZ7 | Day 3–4 | 19.5 | 1.2 | 19.6±6.42 |
| SZ8 | Day 3–4 | 47.6 | 0.5 | 29.3±28.63 |
| SZ9 | Day 2–3 | 31.0 | 0.8 | 36.1±20.9 |
| Mean ± SD |  | 34.2±15.1 |  | 27.4±8.9 |
