# Supplementary figures and images for: Higher amoebic and metronidazole resistant forms of Blastocystis sp. seen in schizophrenic patients
Source: Parasit Vectors. 2022 Sep 5;15:313. doi: 10.1186/s13071-022-05418-0 (PMC9446727; doi:10.1186/s13071-022-05418-0)

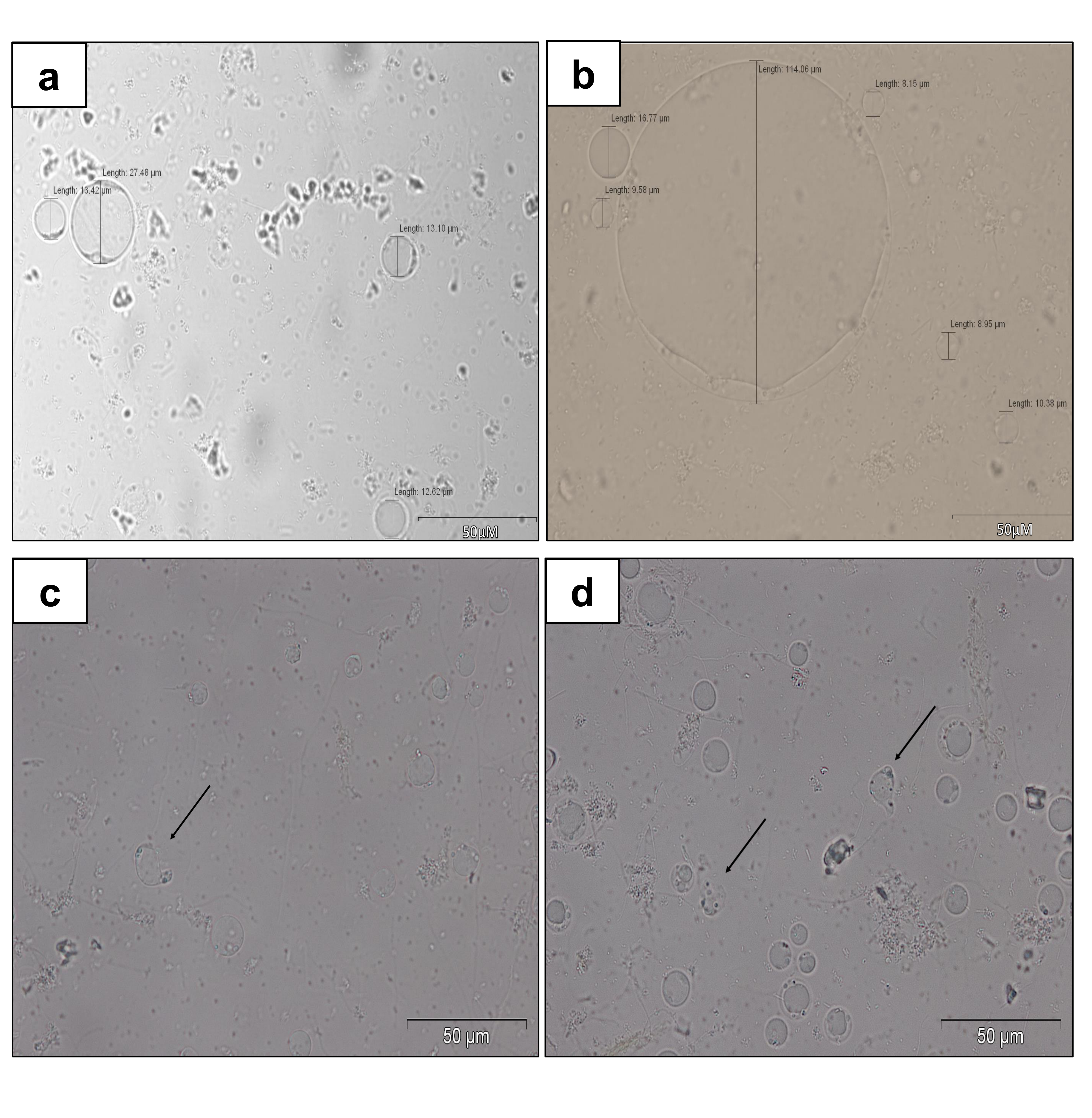

Supplement: Supplementary file 3 — Additional file 3: Fig. S1. Size variation observed among Blastocystis sp. isolated from a non-schizophrenic and b schizophrenic individuals. c, d Amoebic forms observed among Blastocystis sp. isolated from schizophrenic patient (SZ6). Arrows indicates amoebic forms, and all images are viewed at 400× magnification. [file 13071_2022_5418_MOESM3_ESM.tif]

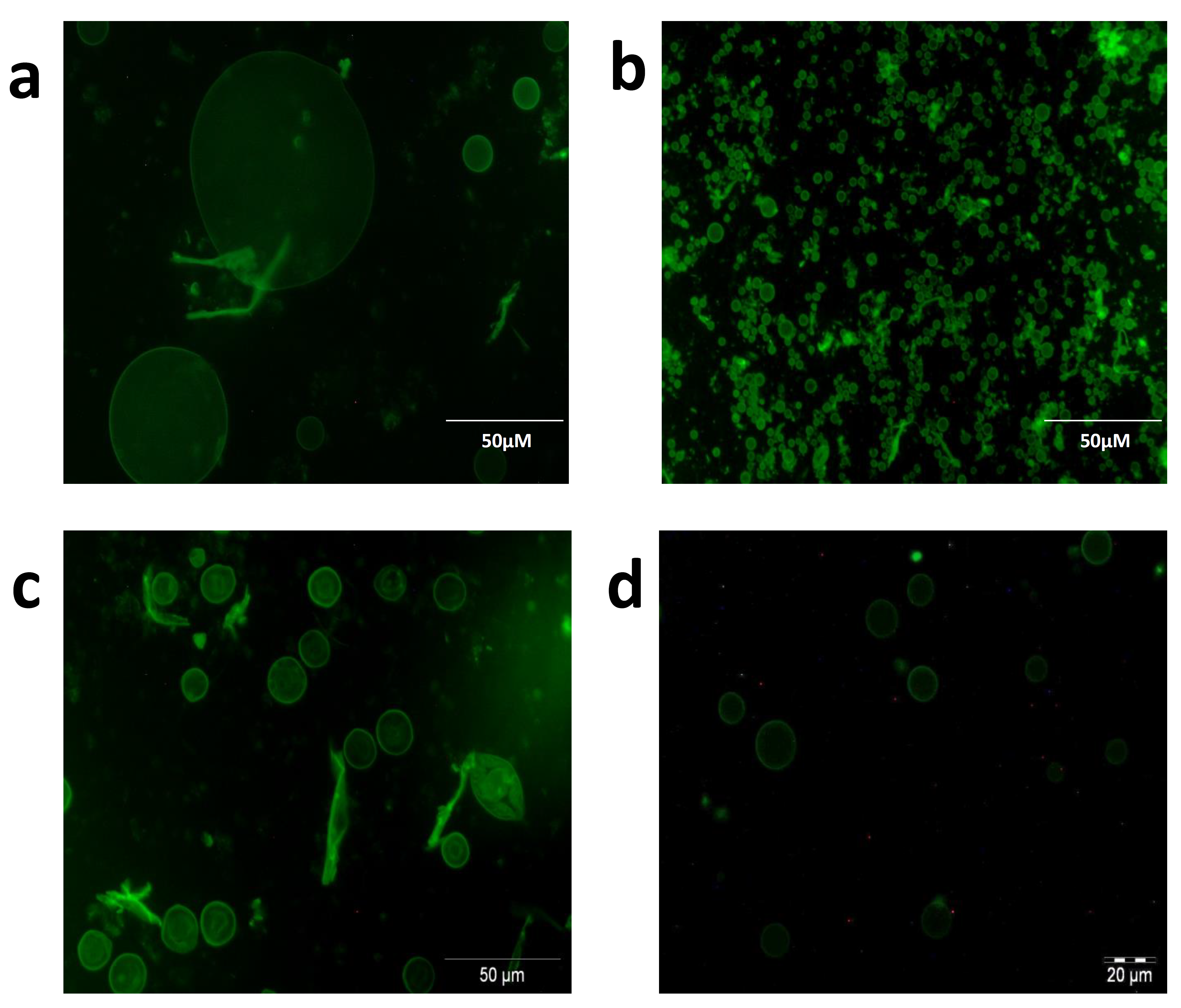

Supplement: Supplementary file 5 — Additional file 5: Fig. S2. Microscopic view of Blastocystis sp. stained with FITC-labelled Concanavalin A. a Schizophrenic isolates with AFU 2+ fluorescence (400×) and b schizophrenic isolates with AFU+3 fluorescence (100×). c Non-schizophrenic isolates with AFU 2+ fluorescence (400×) and d non-schizophrenic isolates with AFU +1 and AFU 0 fluorescence (400×). AFU 0: no fluorescence, AFU 1: + weak intensity, AFU 2+: medium strong, AFU 3+: strong intensity (percentage of reactive forms). [file 13071_2022_5418_MOESM5_ESM.tif]
