# Supplementary material for: Higher amoebic and metronidazole resistant forms of Blastocystis sp. seen in schizophrenic patients
Source: Parasit Vectors. 2022 Sep 5;15:313. doi: 10.1186/s13071-022-05418-0 (PMC9446727; doi:10.1186/s13071-022-05418-0)
Supplement: Supplementary file 4 — Additional file 4: Table S3. Intensity of fluorescence and percentage of fluorescent forms of Blastocystis sp. labelled with FITC-labelled lectins. [file 13071_2022_5418_MOESM4_ESM.docx]

**Table S3:** Intensity of fluorescence and percentage of fluorescent forms of *Blastocystis* sp. labelled with FITC-labelled lectins

|  | FITC-ConA (2 mg/ml) |
| --- | --- |
| Isolates | Fluorescence intensity (% fluorescent forms) |
| **Non-Schizophrenic** |  |
| NS1 | +3 (56%) |
| NS2 | +3 (62%) |
| NS3* | +3 (75%) |
| NS4* | +3 (99%) |
| NS5 | +1 (80%) |
| **Schizophrenic** |  |
| SZ1 | +3 (46%) |
| SZ2 | +3 (62%) |
| SZ3 | +3 (68%) |
| SZ4 | +2 (72%) |
| SZ5* | +3 (92%) |
| SZ6 | +2 (76%) |
| SZ7 | +3 (65%) |
| SZ8 | +2 (52%) |
| SZ9* | +3 (60%) |

*Indicates agglutination/cells clumping
